# The Benefit of Bone Health by Drinking Coffee among Korean Postmenopausal Women: A Cross-Sectional Analysis of the Fourth & Fifth Korea National Health and Nutrition Examination Surveys

**DOI:** 10.1371/journal.pone.0147762

**Published:** 2016-01-27

**Authors:** Eunjoo Choi, Kyung-Hyun Choi, Sang Min Park, Doosup Shin, Hee-Kyung Joh, Eunyoung Cho

**Affiliations:** 1 Department of Family Medicine, Seoul National University Hospital, Seoul National University College of Medicine, 101, Daehak-ro, Jongno-gu, Seoul 110–744, South Korea; 2 Center for Health Promotion & Cancer Prevention, Dongnam Institute of Radiological & Medical Sciences, Jwadong-gil 40, Jangan-eup, Gijang-gun, Busan 619–953, South Korea; 3 Department of Biomedical Sciences, Seoul National University College of Medicine, 28 Yunkeon-dong, Jongro-gu, Seoul 110–744, South Korea; 4 Department of Education and Research, Seoul National University Hospital, 101, Daehak-ro, Jongno-gu, Seoul 110–744, South Korea; 5 Department of Medicine, Seoul National University College of Medicine, 28 Yunkeon-dong, Jongro-gu, Seoul 110–744, South Korea; 6 Department of Family Medicine, Seoul National University Health Service Center, Daehak-dong, Gwanak-gu, Seoul 152–742, South Korea; 7 Channing Division of Network Medicine, Department of Medicine, Brigham and Women's Hospital, Harvard Medical School, 181 Longwood Ave., Boston, MA 02115, United States of America; 8 Department of Dermatology, The Warren Alpert Medical School of Brown University, 339 Eddy St, Providence, RI 02903, United States of America; UAMS, UNITED STATES

## Abstract

**Purpose:**

Although the concern about coffee-associated health problems is increasing, the effect of coffee on osteoporosis is still conflicting. This study aimed to determine the relationship between coffee consumption and bone health in Korean postmenopausal women.

**Methods:**

A population-based, cross-sectional study was performed using a nationally representative sample of the Korean general population. All 4,066 postmenopausal women (mean age 62.6 years) from the fourth and fifth Korean National Health and Nutrition Examination Survey (2008–2011), who completed the questionnaire about coffee consumption and had data of dual-energy X-ray absorptiometry (DXA) examination. Bone mineral density (BMD) was measured using DXA at the femoral neck and lumbar spine and osteoporosis was defined by World Health Organization T-score criteria in addition to self-report of current anti-osteoporotic medication use.

**Results:**

After adjusting for various demographic and lifestyle confounders (including hormonal factors), subjects in the highest quartile of coffee intake had 36% lower odds for osteoporosis compared to those in the lowest quartile (Adjusted odds ratio [aOR] = 0.64; 95% confidence interval [CI], 0.43–0.95; P for trend = 0.015). This trend was consistent in osteoporosis of lumbar spine and femoral neck (aOR = 0.65 and 0.55; P for trend = 0.026 and 0.003, respectively). In addition, age- and body mass index (BMI)-adjusted BMD of the femoral neck and lumbar spine increased with higher coffee intake (P for trend = 0.019 and 0.051, respectively).

**Conclusions:**

Coffee consumption may have protective benefits on bone health in Korean postmenopausal women in moderate amount. Further, prospective studies are required to confirm this association.

## Introduction

Coffee is one of the most popular beverages in the world, as well as the most widely traded agricultural commodity [1]. World coffee production in 2013/14 was at a record high of 150.5 million bags and global stocks have been rising over the last several years [2]. Asia has experienced the most dynamic growth in coffee consumption in the world, growing by an average rate of 4% per annum since 1990 and increasing to 4.9% of the total population since 2000 [3]. Accordingly, concern about coffee-associated health problems is increasing. It has been shown that coffee might have the protective benefit of occurrence of some diseases such as type 2 diabetes mellitus, cirrhosis, Parkinson’s disease, and cardiovascular disease [4–6]. On the other hand, several studies have found a harmful effect of coffee on serum cholesterol and homocysteine concentrations [7, 8]. Many of the previous studies focused on the potential effects of caffeine or caffeine-containing beverages (including green tea, cola, etc.) on bone metabolism because caffeine is considered to be a major component of coffee [9–11]. This approach, however, may not be optimal to evaluate the role of coffee on bone mineral density (BMD) because coffee contains not only caffeine but also other bioactive substances that may affect BMD.

Coffee is interesting with respect to osteoporosis etiology because of its complex ingredients, several of which has been shown in experimental studies to have a potential to alter the risk of osteoporosis through meaningful biological mechanisms [1, 11–13]. Results from epidemiological studies investigating the relation between coffee consumption and BMD in both women [14] and men [15–17] have been conflicting, which might be explained by differences in sample size, method of data collection, and amount of coffee consumed. Previous studies demonstrated that the adverse effect of coffee on calcium balance could be explained by a decrease in calcium absorption and an increase in the excretion of urinary calcium and fecal calcium [5]. They, however, did not take into account the fact that intestinal absorption and urinary excretion of calcium are estrogen dependent [6–8]. It is important to evaluate the effect of coffee on bone health in postmenopausal women since they are especially vulnerable to bone loss due to estrogen deficiency [18, 19]. Furthermore, Asian women have lower bone mineral density than Caucasians, and the prevalence of osteoporosis continues to increase in the aging population [20]. Nevertheless, the characteristics of osteoporosis in Asia are less defined than that in Western countries, and the effect of coffee on BMD is still unknown. Considering the substantial increase of coffee consumption, it is necessary to investigate the relation between coffee and bone health in Asian countries. Therefore, the present study was aimed to explore the relation between coffee consumption and BMD and the risk of osteoporosis in Korean postmenopausal women. We hypothesized that other components other than caffeine from coffee would have a good protective influence on bone health in Korean postmenopausal women.

## Materials and Methods

### Subjects and Data Collection

This study used data obtained from the Korean National Health and Nutritional Examination Survey (KNHANES) of non-institutionalized Korean civilians, which was conducted by the Korean Ministry of Health and Welfare from 2008–2011. This survey was a nationwide representative study that used a stratified, multistage probability sampling design for the selection of household units. Detailed descriptions of the methods for survey have been described elsewhere [21]. In this study, data collected from participants who completed both the Health and the Nutrition portions of the Survey were used. During the period (2008–2011), a total of 37,753 participants (17,195 men and 20,558 women) completed the survey.

**Fig 1:**
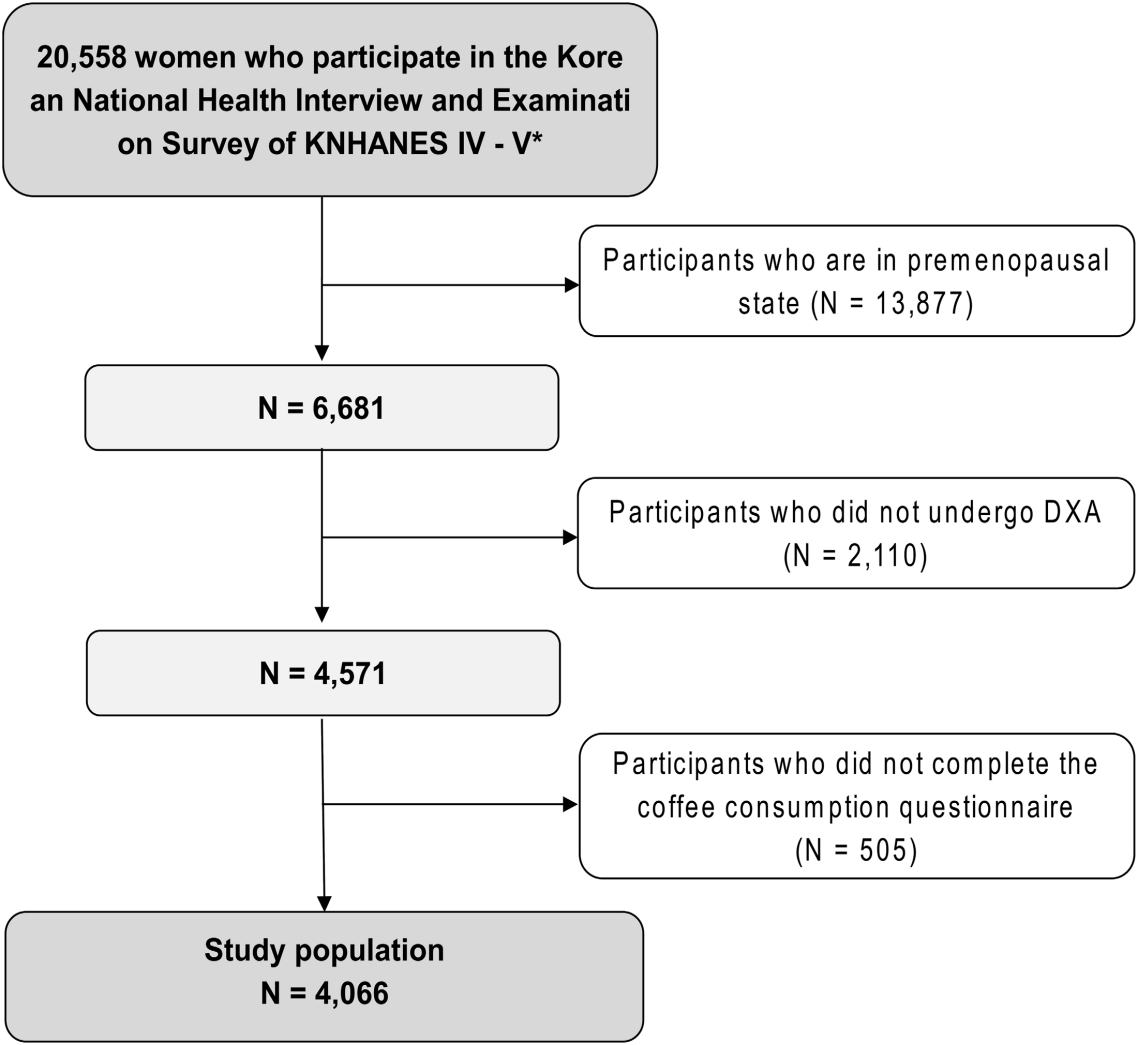
The study population framework. ^a^ 2008–2011 The Fourth and Fifth Korean National Health and Nutritional Examination Survey.

We excluded men (n = 17,195) and premenopausal women (n = 13,877). Among the 6,681 postmenopausal women, participants without dual-energy X-ray absorptiometry (DXA) examination data (n = 2,110) were excluded. After additional exclusion of participants who did not complete the questionnaire about coffee consumption (n = 505), 4,066 women were finally included in this study (Fig 1). All the participants provided informed consent. No ethical approval of our Institutional Review Board was required since this survey data were publicly available.

### Lifestyle factors and measurements of anthropometric parameters

Questions concerning demographic and lifestyle factors (including smoking habits, alcohol consumption, physical activity, education level, monthly income, dietary intake of coffee, tea, and calcium) were completed by all the participants. Smoking status was categorized as current smoker (someone who smoked cigarettes daily), ex-smoker (someone who smoked in the past but does not smoke cigarettes currently), and never smoker (someone who has never smoke a cigarette). Based on how often participants consumed any type of alcohol, alcohol consumption was categorized as less than 3 standard drinks [StDs] per occasion, and equal to or greater than 3 [StDs] per occasion) [22, 23]. Physical activity was assessed by using the international physical activity questionnaire (IPAQ), and categorized as following: Participants who practice more than 20 minute of vigorous activity at least three times a week, or more than 30 minute of light/moderate activity at least five times a week. [24]. With regard to educational background status, the participants were divided into elementary school graduate, middle or high school graduate, and college graduate or above. Age at menarche, age of menopause, and use of hormonal agent of the participants were also asked.

Anthropometric measurements were taken by well-trained examiners. Height was measured by 0.1 cm unit and weight was measured by 0.1 kg unit, and body mass index (BMI) was calculated as the ratio of weight (kg) to height^2^ (m^2^). Dietary calcium intake was assessed using the 24-hour diet recall method, analyzed by CAN-Pro software 3.0 (Korean Nutrition Society, Seoul, Korea). A 24-hour recall is methodologically convenient and feasible to assess dietary intakes in a large population, and has been used in previous population-based researches [10].

### Measurements of BMD

The KNHANES Osteoporosis survey was a large-scale BMD survey in which accurate and reliable data were gathered by trained osteoporosis examination surveyors from Korean government [25]. Femoral neck and lumbar spine [L1 –L4] BMD (g/cm^2^) were measured by DXA scan (DISCOVERY-W fan-beam densitometer, Hologic Inc., Santa Clara, CA). The diagnosis of osteoporosis was made using WHO T-score criteria (T-score ≤ −2.5) and the participants who were taking prescription medications (e.g., bisphosphonate, raloxifene, hormonal agents, etc.) were considered as having osteoporosis, since the medication could increase their BMD. During the KNHANES IV–V survey, only osteoporosis patients with BMD T-scores < −3.0 or with X-ray-confirmed fractures could receive prescriptions of hormonal and non-hormonal anti-osteoporotic drugs under National Health Insurance coverage in Korea [26]. Although KNHANES IV–V included self-reported fracture data, a past medical history of fracture was not used to define osteoporosis, due to the following reasons. First, it was difficult to assess the association between coffee consumption and osteoporotic fracture since it was cross-sectional study. Second, KNHANES IV–V included neither confirmatory imaging tests nor means of distinguishing between low- and high-energy fractures during history taking.

### Assessment of coffee consumption

The frequency of coffee consumption was investigated by the questionnaire, since the self-administered food frequency questionnaire for coffee consumption was a validated method in Asian population [27]. There were nine categories of the frequency of coffee consumption in KNHANES: 3 cups a day, 2 cups a day, 1 cups a day, 4–6 cups a week, 2~3 cups a week, 1 cups a week, 2–3 cups a month, 1 cups a month, 6–11 cups a year, almost no drinking. Taking into account the distribution of the participants, the participants were re-categorized as four groups as following: non-consumers (n = 872) included participants who rarely drink coffee (less than once a month); participants (n = 785) who drink coffee irregularly (more than once a month and less than once a day); participants (n = 1,421) who drink coffee once a day; participants (n = 988) who drink coffee more than once a day (twice or thrice a day). In this study, the upper limit of the frequency was three times a day owing to the limitation of the questionnaire.

### Statistical Analysis

We used a weighted population sample to reflect the sampling method and response rate. Clinical and biochemical characteristics among groups were compared according to coffee consumption using univariate analysis, when the variables were continuous and categorical, respectively. The estimated proportions and standard errors for osteoporosis (whole body site) were calculated according to the frequency of coffee consumption. Unadjusted and multivariate logistic regression analysis was conducted to estimate crude and adjusted odd ratios (aORs) and 95% confidence intervals (CI) for the risk of osteoporosis according to the coffee consumption categories, using the lowest intake category as a reference. ORs were primarily calculated following adjustment for age and BMI. Current model 1 was adjusted for behavioral (including nutrition) factors in addition to age and BMI. For the behavioral factors, smoking status, alcohol consumption, physical activity, serum vitamin D level, and nutritional factors (such as dietary intake of calcium and regular tea consumption) were included. Current model 2 was adjusted for socioeconomic status factors (including monthly household income and education level) and hormonal factors (including age at menopause and use of hormonal agent) in addition to all variables in current model 1. A trend test was carried out by modeling the main independent values as continuous variables. With regard to the conditions affecting bone metabolism (chronic disease, such as cardiovascular disease, cerebrovascular disease, cancer), all participants answered the questionnaire whether they had been diagnosed and currently treated by physicians. Using the data, we grouped the participants into two categories: (1) having any of comorbidity which is known to be related to osteoporosis (n = 506), (2) having no comorbidity (n = 3,560), and adjusted comorbidity as a covariate in addition to current model 2 (data not shown). Further, we performed subgroup analysis among the participants without any comorbidity. To investigate the relationship between coffee consumption and BMD, least-squares mean of whole body, femur neck, lumbar spine of BMD stratified by coffee consumption. And, multiple linear regression model adjusted for age, BMI was used for a linear trend in BMD.

All results were considered significant if p < 0.05. All analyses were performed using STATA statistical software release 12.1 (STATA Corp., College Station, TX). Using “svy” commands, complex sampling weights were accounted and this enabled the results to represent the entire national adult population.

## Results

### The characteristics of the study participants

The general characteristics according to the frequency of coffee consumption are presented in Table 1. Over the half of the study population (n = 2,250) drank coffee at least once a day, and younger participants were more likely to have more coffee. Mean calcium intake was 417 mg/day, which was much less than the recommended amount of over 1200 mg/day for postmenopausal women [28]. The prevalence of osteoporosis was 40.2% (n = 1,635) and 281 (15%) underwent hormone replacement therapy (HRT). All the demographic and lifestyle factors were shown to be significant different by the presence of osteoporosis, except calcium supplement and age of menarche. Participants with osteoporosis were older than participants without osteoporosis (mean age, 69 and 60, respectively) and less smoke, less drink alcohol, and less exercise. In addition, they were less educated and had less income than participants without osteoporosis.

**Table 1:** The characteristics of participants according to the coffee consumption (lumbar spine or femoral neck T-score ≤ −2.5, or taking anti-osteoporotic medications).

Variable	Categories of coffee consumption, cup/d (n^a^ = 4,066)				
	None	<1	1	2	
	(n* = 872)	(n* = 785)	(n* = 1,421)	(n* = 988)	P value
Number* (%)					
Age, mean (SD), years	66.4(8.9)	64.1(9.1)	64.1(9.3)	60.7(8.8)	0.00
BMI, mean (SD), kg/m²	23.9(3.2)	24.2(3.3)	204.2(3.1)	24.493.3)	0.010
Smoking, n (%)					0.014
None	829(96.6)	745(95.3)	1331(94.3)	866(88.0)	
Ex-smoker	24(2.8)	19(2.4)	38(2.7)	27(2.7)	
Current smoker	14(1.6)	18(2.3)	42(3.0)	91(9.3)	
High-risk drinking, n (%)	14(1.6)	23(2.9)	48(3.40)	58(5.9)	0.005
Regular exercise, n (%)	155(17.8)	155(19.8)	316(22.4)	249(25.2)	0.006
Calcium intake, mean (SD), mg/day	399.1(311.4)	422.2(289.9)	418(454.2)	438.2(315.6)	0.203
Calcium supplement, n (%)	219(25.1)	165(21.0)	328923.1)	200(20.2)	0.089
Education level, n (%)					0.000
≥ College	38(4.4)	41(5.2)	60(4.3)	58(5.9)	
Middle/high	175(20.3)	235(50.0)	428(30.3)	361(36.8)	
≤ Elementary	649(75.3)	507(64.8)	923(65.4)	562(57.3)	
Monthly income, n (%), thousand won					0.000
≤ 1000	439(50.8)	334(43.20	572(41.0)	335(34.5)	
1010–3000	259(30.0)	251(32.4)	479(34.3)	361(37.4)	
≥ 3010	166(19.2)	189(24.4)	345(24.7)	275(28.3)	
Vitamin D level,mean (SD), ng/ml	19.3(7.3)	19.0(7.3)	18.8(7.1)	18.5(6.9)	0.247
Regular tea con sumption, n (%)	40(4.6)	2693.30	148(10.4)	129(13.1)	0.0000.
Age of menarche, mean (SD), years	16.0(1.8)	15.9(1.9)	15.9(1.9)	15.8(2.0)	0.121
Age of menopause, mean (SD), years	48.9(4.6)	48.7(5.0)	49.0(4.5)	49.1(5.2)	0.682
HRT, n (%)	550(63.1)	412(52.5)	754(53.1)	489(49.5)	0.153

Data are number (%) or mean ± standard deviations (SD). Abbreviations: BMI: body mass index; HRT, hormone replacement therapy. All data are weighted to the residential population of Korea.

* n: unweighted sample size.

### ORs of osteoporosis according to coffee consumption

Coffee consumption was inversely associated with osteoporosis (Table 2). In an unadjusted analysis, OR for osteoporosis in the highest quintile of coffee intake with the lowest was 0.40 (95% CI, 0.31–0.51), and an inverse association between coffee consumption and osteoporosis was seen (P for trend < 0.001). This trend was maintained after adjusting for potential confounders, and in current model 2, subjects in the highest coffee intake quartile had 36% lower odds of having osteoporosis compared to those in the lowest quartile (aOR = 0.64; 95% CI, 0.43–0.95; P for trend = 0.015). In addition, when we stratified the population by age (< 65 years vs. ≥ 65 years), the trend remained consistent, though it was not statistically significant. The aOR for osteoporosis in the highest coffee consumer comparing to non-consumer was 0.59 (P for trend = 0.058) among the participants under 65 and 0.74 (P for trend = 0.139) among the participants with age of 65 and over (data not shown).

**Table 2:** Coffee consumption and osteoporosis (Lumbar spine or femoral neck T-score < −2.5, or taking anti-osteoporotic medications) according to the body site.

	Categories of coffee consumption, cup/d (n^a^ = 4,066)				
	None	<1	1	2	*P for trend*
	(n* = 872)	(n* = 785)	(n* = 1,421)	(n* = 988)	
**Femoral neck or lumbar spine osteoporosis**^†^ **(n = 1,653)**					
Estimated proportion, % (SE)	50.1 (2.1)	37.3 (2.1)	35.1 (1.6)	28.6 (1.8)	
Unadjusted OR (95% CI)	1(Ref)	0.59 (0.46,0.75)	0.54 (0.43,0.66)	0.40 (0.31,0.51)	<0.001
Age and BMI adjusted OR (95% CI)	1(Ref)	0.78 (0.58,1.04)	0.69 (0.54,0.89)	0.80 (0.61,1.07)	0.071
Current model 1^‡^	1(Ref)	0.75 (0.56,1.00)	0.67 (0.52,0.86)	0.79 (0.59,1.06)	0.067
		0.059	0.002	0.122	
Current model 2^§^	1(Ref)	0.79 (0.53,1.17)	0.67 (0.47,0.95)	0.64 (0.43,0.95)	0.015
		0.198	0.020	0.032	
**Lumbar spine osteoporosis (n = 1,350)**					
Estimated proportion, % (SE)	41.0 (2.1)	30.1 (2.0)	27.7 (1.4)	23.3 (1.7)	
Unadjusted OR (95% CI)	1(Ref)	0.62 (0.48,0.80)	0.55 (0.44,0.69)	0.44 (0.34,0.56)	<0.001
Age and BMI adjusted OR (95% CI)	1(Ref)	0.78 (0.59,1.03)	0.68 (0.53,0.87)	0.76 (0.57,1.01)	0.079
Current model 1^‡^	1(Ref)	0.79 (0.59,1.05)	0.72 (0.55,0.93)	0.81 (0.60,1.10)	0.112
		0.109	0.013	0.192	
Current model 2^§^	1(Ref)	0.82 (0.56,1.20)	0.75 (0.54,1.05)	0.65 (0.44,0.96)	0.026
		0.255	0.081	0.038	
**Femoral neck osteoporosis (n = 1,035)**					
Estimated proportion, % (SE)	35.6 (2.1)	23.8 (1.8)	23.0 (1.4)	14.9 (1.3)	
Unadjusted OR (95% CI)	1(Ref)	0.56 (0.43,0.73)	0.54 (0.43,0.68)	0.32 (0.24,0.42)	<0.001
Age and BMI adjusted OR (95% CI)	1(Ref)	0.75 (0.55,1.02)	0.70 (0.53,0.91)	0.64 (0.46,0.87)	0.003
Current model 1^‡^	1(Ref)	0.69 (0.51,0.94)	0.63 (0.48,0.83)	0.56 (0.40,0.78)	<0.001
		0.017	0.001	0.001	
Current model 2^§^	1(Ref)	0.73 (0.49,1.09)	0.63 (0.44,0.90)	0.55 (0.36,0.84)	0.003
		0.099	0.007	0.006	

Abbreviations: OR, odds ratio; CI, confidence interval; BMI, body mass index. All data are weighted to the residential population of Korea.

* n; unweighted sample size.

^†^ The definitions of osteoporosis were calculated using World Health Organization (WHO) T-score of the lumbar spine or femoral neck (T-score ≤ −2.5), and included those taking anti-osteoporotic medications.

^‡^ Current model 1 is adjusted for behavioral(including diet) factors in addition to age and BMI.

^§^ Current model 2 is adjusted for socioeconomic status factors (including monthly household income and education level) and hormonal factors in addition to all variables in current model 1.

Further, although we conducted the multivariate regression analysis only in participants without any comorbidity, overall trends and results were still maintained (Table 3). The trend was not change when we adjusted comorbidity as a covariate in addition to current model 2 regardless of part of the body (P for trend = 0.013, 0.018, and 0.007, respectively, data not shown).

**Table 3:** Coffee consumption and osteoporosis (Lumbar spine or femoral neck T-score < −2.5, or taking anti-osteoporotic medications) by the body site, among the participants without any comorbidity.

	Categories of coffee consumption, cup/d (n^a^ = 3,560)				
	None	<1	1	2	*P for trend*
	(n* = 732)	(n* = 669)	(n* = 1,260)	(n* = 899)	
**Femoral neck or lumbar spine osteoporosis**^†^ **(n = 1,422)**					
Estimated proportion, % (SE)	51.4 (2.3)	38.5 (2.3)	34.8 (1.7)	28.3 (1.9)	
Unadjusted OR (95% CI)	1(Ref)	0.59 (0.45,0.77)	0.50 (0.40,0.63)	0.37 (0.29,0.48)	<0.001
Age and BMI adjusted OR (95% CI)	1(Ref)	0.82 (0.60,1.12)	0.65 (0.50,0.85)	0.76 (0.56,1.03)	0.029
Current model 1^‡^	1(Ref)	0.78 (0.57,1.08)	0.63 (0.48,0.83)	0.76 (0.55,1.04)	0.037
		0.059	0.002	0.122	
Current model 2^§^	1(Ref)	0.81 (0.53,1.25)	0.63 (0.43,0.91)	0.63 (0.41,0.96)	0.013
		0.198	0.020	0.032	
**Lumbar spine osteoporosis (n = 1,170)**					
Estimated proportion, % (SE)	41.9 (2.3)	31.4 (2.2)	27.3 (1.6)	22.8 (1.7)	
Unadjusted OR (95% CI)	1(Ref)	0.64 (0.49,0.84)	0.52 (0.41,0.66)	0.41 (0.31,0.54)	<0.001
Age and BMI adjusted OR (95% CI)	1(Ref)	0.84 (0.62,1.13)	0.65 (0.50,0.84)	0.72 (0.53,0.97)	0.008
Current model 1^‡^	1(Ref)	0.81 (0.59,1.11)	0.68 (0.52,0.90)	0.77 (0.56,1.07)	0.061
		0.109	0.013	0.192	
Current model 2^§^	1(Ref)	0.82 (0.54,1.24)	0.71 (0.49,1.01)	0.63 (0.42,0.95)	0.018
		0.255	0.081	0.038	
**Femoral neck osteoporosis (n = 906)**					
Estimated proportion, % (SE)	36.5 (2.3)	24.3 (2.0)	23.0 (1.5)	15.1 (1.4)	
Unadjusted OR (95% CI)	1(Ref)	0.56 (0.42,0.74)	0.52 (0.42,0.67)	0.31 (0.23,0.42)	<0.001
Age and BMI adjusted OR (95% CI)	1(Ref)	0.79 (0.57,1.10)	0.69 (0.52,0.92)	0.63 (0.45,0.89)	0.004
Current model 1^‡^	1(Ref)	0.72 (0.51,1.01)	0.62 (0.46,0.83)	0.57 (0.40,0.81)	0.001
		0.017	0.001	0.001	
Current model 2^§^	1(Ref)	0.78 (0.51,1.20)	0.62 (0.42,0.89)	0.59 (0.38,0.92)	0.007
		0.099	0.007	0.006	

Abbreviations: OR, odds ratio; CI, confidence interval; BMI, body mass index. All data are weighted to the residential population of Korea.

* n; unweighted sample size.

^†^ The definitions of osteoporosis were calculated using World Health Organization (WHO) T-score of the lumbar spine or femoral neck (T-score ≤ −2.5), and included those taking anti-osteoporotic medications.

^‡^ Current model 1 is adjusted for behavioral(including diet) factors in addition to age and BMI.

^§^ Current model 2 is adjusted for hormonal factors in addition to all variables in current model 2.

### Adjusted mean of BMD according to coffee consumption

**Fig 2:**
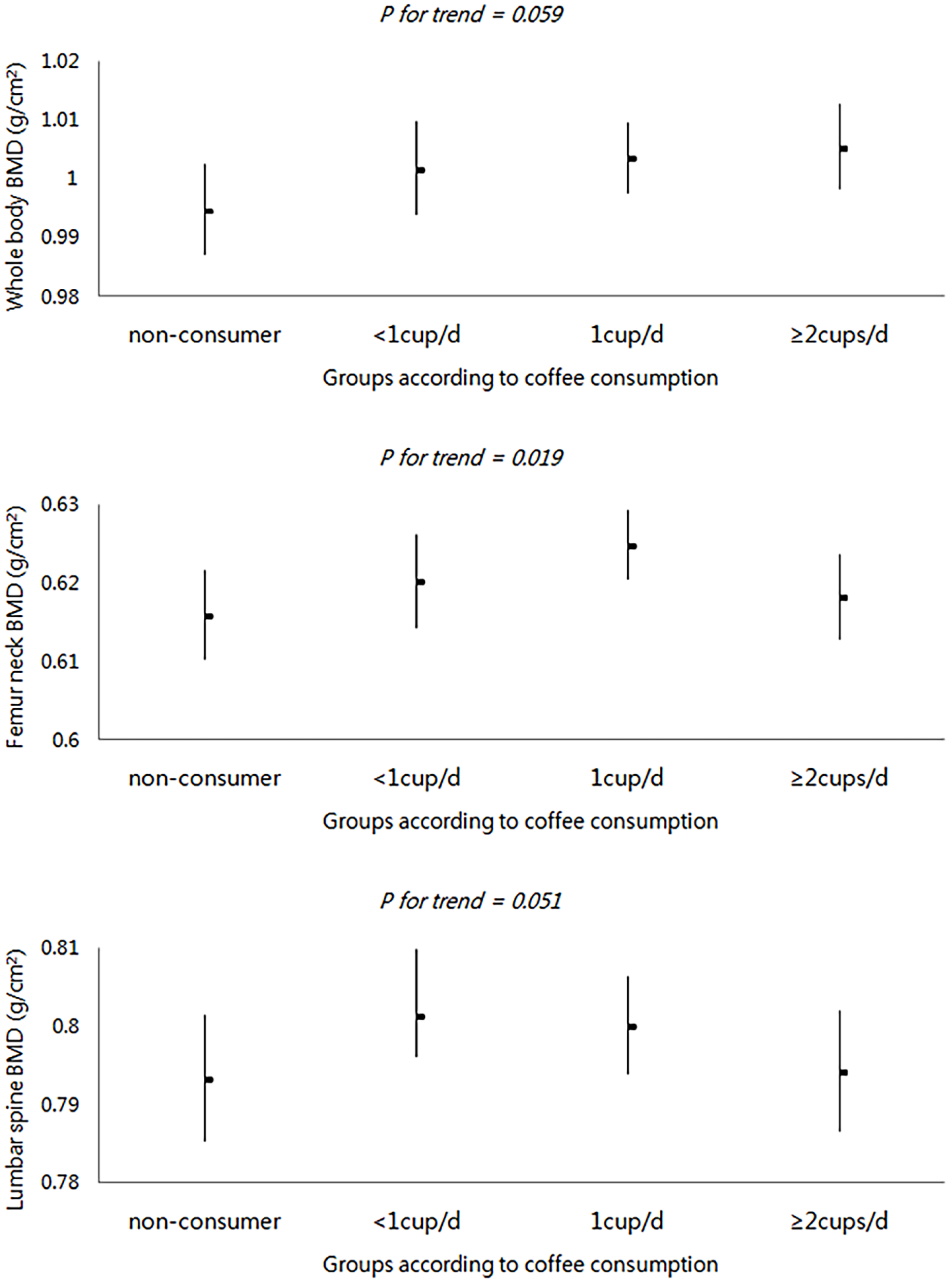
Least-squares means of the bone mineral density, adjusted for age and BMI, according to coffee consumption. Abbreviations: BMD, bone mineral density; BMI, body mass index. Least-squares mean of whole body, femur neck, lumbar spine of BMD stratified by coffee consumption. Multiple linear regression model adjusted for age, BMI was used for a linear trend in BMD.

The relationship between coffee consumption and BMD is presented in Fig 2. Age- and BMI-adjusted whole body BMD increased with higher coffee consumption (P for trend = 0.059). The same results were found for BMD of the femoral neck and lumbar spine (P for trend = 0.019 and 0.051, respectively). After stratification by age (< 65 years vs. ≥ 65 years), the positive association between coffee consumption and BMD did not change, although the significance was attenuated (data not shown).

## Discussion

In this study, we found that participants in the highest quartile of coffee intake had the lowest odds for osteoporosis compared to those in the lower coffee consumption. The result was not different when analyzing the relation between coffee consumption and BMD linearly. As coffee consumption increased, so did the BMD of femoral neck and lumbar spine. Age- and BMI-adjusted BMD of femoral neck and lumbar spine also increased with higher of coffee consumption.

Previous prospective studies of elderly populations yielded conflicting results regarding the effect of coffee on bone loss [14, 15, 29, 30]. In prior studies showing a positive association, risk of osteoporosis (osteoporosis-induced fracture) was significantly increased when more than 4 cups/day of coffee (330 mg of caffeine, equivalent to 600 ml of coffee) was consumed (relative risk [RR], 1.2–1.9) [11, 14, 30]. This is quite a significant amount of coffee considering that the average amount of coffee consumption is merely 0.7 cup/day in the Korean population [29]. Further, the effect might be explained in part by an inverse relationship between consumption of milk and caffeine-containing beverages. The negative effect of caffeine on calcium absorption or skeletal fragility is enough to be compensated by small amount of milk.[9]. In this study, however, we found an inverse relationship between coffee consumption and osteoporosis, even after adjusting for both dietary calcium intake and calcium supplement use. This result was consistent with several other studies that demonstrated that coffee consumption was not harmful to bone health [15, 31, 32].

This may be due to the effect of the other biochemical material other than caffeine from coffee component, we speculate the following as plausible explanations: (1) estrogenic effect of coffee; (2) antioxidant effect of coffee; and (3) anti-inflammatory properties of coffee. First, estrogenic effects of coffee can modulate the risk of osteoporosis. In a recent study, Lee et al. showed a different effect of coffee in premenopausal women and postmenopausal women [31]. The risk of osteoporotic fracture was higher in premenopausal women (RR = 1.29; 95% CI, 1.05–1.57) but lower in postmenopausal women (RR = 0.98; 95% CI, 0.76–1.25). This could be due to the abundant endogenous estrogen in premenopausal women. Loss of estrogen at menopause is considered the greatest potential risk factor for bone loss in women; therefore, HRT including estrogen is approved for prevention of bone loss in menopausal women [33]. Coffee is a chemically complex beverage containing many compounds that can affect the estrogen level [34–37]. In an experimental study, Kitts showed that coffee contains biologically active constituents that can bind to cytoplasmic estrogen receptor protein in vitro and induce uterine cellular responses in mice in vivo [34]. Moreover, Allred et al. demonstrated that trigonelline, a natural constituent of coffee accounting for ∼1% of dry matter in roasted beans, can function as an estrogen receptor (ER) agonist in estrogen-responsive breast cancer cells and that the compound results in transcriptional activation of the ER, which alters the gene expression of downstream target genes, and finally exerts potential benefits for human health [35, 36]. Recently, Schliep observed that higher caffeine intake (≥ 200 mg/day) was associated with increased estradiol concentrations among Asian women, whereas it was inversely associated among whites [3].

Second, antioxidant effects of coffee can reduce the risk of osteoporosis. Researchers found that a typical serving of coffee contains more antioxidants than typical servings of grape juice, blueberries, raspberries, and oranges [38]. Polyphenols are varied molecules containing approximately 5,000 species including flavonoids (e.g., catechins in tea, isoflavones in beans, lutein in vegetables, and anthocyanins in fruit) and non-flavonoids (chlorogenic acids in coffee). Chlorogenic acid in coffee is shown to have powerful inhibitory effects on osteoclastogenesis by suppressing the expression of NFATc1, a key transcription factor for the induction of osteoclastogenesis [39].

Third, anti-inflammatory effects of coffee can modulate the risk of osteoporosis. Kahweol, a coffee-specific diterpene, was proven to have significant anti-inflammatory effects in vivo, which might be due to the inhibition of inducible nitric oxide synthase and cyclooxygenase-2 (COX-2) expression in the inflammatory sites [40, 41]. Previous studies suggested that several drugs with anti-inflammatory effects, including COX-2 selective non-steroidal anti-inflammatory drugs such as aspirin and bisphosphonates, are associated with higher BMD [42]. On the basis of these findings, we could assume that the anti-inflammatory effect of coffee ingredients might be one reason for the association between coffee consumption and bone health. These materials of coffee components can affect bone health, and further studies may be needed to assess the effects of coffee on bone health with specific investigation into the ingredients of the various coffees that are consumed.

Our study had several limitations, including that the contents or brewing methods of the coffee was not fully evaluated. Although the participants might have consumed different types of coffee, the preference might not be significantly different since they are the same sex and within a similar age group. Further, since the data was completed by using a food frequency questionnaire, individual’s habitual diet could not be fully assessed. It should be noted that food frequency questionnaire of this data did not evaluate brewing methods or additional ingredient of coffee (milk, cream powder, sugar-contained or decaffeinated, etc) In addition, as the range of coffee consumption is relatively narrow owing to the limitation of the questionnaire in KNHANES, the maximal dose of coffee intake was 3 cups/day, whereas other studies have shown the adverse effect of ≥ 4 cups/day of coffee. However, considering a substantial proportion of the Korean population drinks coffee less than once a day, such a high consumption rate could be ignored in this study [43]. So, it cannot be determined the quantitative relationship of coffee consumption and bone health in this data. And this study also couldn’t assess the precise caffeine amount because it was difficult to obtain accurate information on the caffeine content of all beverages. The result suggest proper coffee intake have the benefit on bone health. And, given the cross-sectional nature of the surveys, causal relationships between osteoporosis of postmenopausal women and other independent variables including coffee consumption cannot be determined. Future studies should expand a prospective design to evaluate the effect or mechanism of coffee consumption on bone health among postmenopausal women. To overcome the issue of reverse causality in such a cross-sectional study design, we included several known confounding factors, used age stratification, and performed subgroup analysis. In spite of the limitations, our study is unique because the results were representative of the general population of the entire nation after adjusting for several confounders and focusing on unique characteristics of coffee, not limited to caffeine.

In conclusion, within moderate ranges of consumption, coffee was inversely associated with osteoporosis and directly associated with bone mineral density among Korean postmenopausal women. Caution regarding effect modification by the amount of coffee consumed in this study is warranted because of the narrow range of coffee consumption. Understanding the relation between coffee and bone health in postmenopausal women has substantial implications, both in regard to increasing exposure and to public health concerns about osteoporosis-related fracture. Given this public health implication, further research of how various components of coffee influence the skeletal system, and whether these influences differ by race, is needed.
